# Continuation Versus Interruption of Anticoagulation During Transcatheter Aortic Valve Implantation: A Systematic Review and Meta-Analysis

**DOI:** 10.7759/cureus.76434

**Published:** 2024-12-26

**Authors:** Godfrey Tabowei, Samuel K Dadzie, Rana Muhammad Ahzam, Mian M Rehman, Keron Blair, Ihtisham Habib, Calvin R Wei, Adil Amin

**Affiliations:** 1 Internal Medicine, Texas Tech University Health Sciences Center, Odessa Campus, Odessa, USA; 2 Internal Medicine, Piedmont Athens Regional Medical Center, Athens, USA; 3 Internal Medicine, Combined Military Hospital (CMH) Lahore Medical College and Institute of Dentistry, Lahore, PAK; 4 Cardiology, Combined Military Hospital (CMH) Lahore Medical College and Institute of Dentistry, Lahore, PAK; 5 Medicine, American International School of Medicine, Georgetown, GUY; 6 Internal Medicine, Medical Teaching Institute, Lady Reading Hospital, Peshawar, PAK; 7 Research and Development, Shing Huei Group, Taipei, TWN; 8 Cardiology, Pakistan Navy Station Shifa, Karachi, PAK

**Keywords:** bleeding risk, meta-analysis, oral anticoagulation, perioperative management, transcatheter aortic valve implantation

## Abstract

Transcatheter aortic valve implantation (TAVI) involves complex decisions regarding perioperative anticoagulation, with continuation or interruption of oral anticoagulation presenting distinct risks and benefits. This systematic review and meta-analysis compared the clinical outcomes of these two strategies during TAVI. We conducted a comprehensive literature search across multiple electronic databases, including PubMed, Embase, Cochrane Library, and Web of Science, from inception to November 2024. Three studies with 2,591 patients (1,132 in the continuation group and 1,459 in the interruption group) met the inclusion criteria. The primary outcomes included all-cause mortality, myocardial infarction (MI), stroke, and major bleeding within one month of the procedure. Risk ratios (RRs) with 95% confidence intervals (CIs) were calculated using a random-effects model. Analysis revealed no significant differences between continuation and interruption groups for all-cause mortality (RR: 0.87, 95% CI: 0.53-1.41, *P*-value: 0.56), MI (RR: 0.68, 95% CI: 0.23-1.97, *P*-value: 0.48), stroke (RR: 0.67, 95% CI: 0.42-1.08, *P*-value: 0.10), or major bleeding (RR: 0.93, 95% CI: 0.69-1.26, *P*-value: 0.63). No substantial heterogeneity was observed across studies for any outcome. While continued anticoagulation showed a trend toward lower stroke risk, this difference did not reach statistical significance. The findings suggest that both strategies may be reasonable options, though the limited number of studies and short follow-up duration highlights the need for larger randomized controlled trials (RCTs). Until more definitive evidence emerges, the choice between continuation and interruption of oral anticoagulation during TAVI should be individualized based on patient-specific thromboembolic and bleeding risk factors.

## Introduction and background

Transcatheter aortic valve implantation (TAVI) has emerged as a revolutionary treatment for aortic valve diseases, particularly in high-risk patients [1]. With over one million procedures performed globally and success rates exceeding 90% in most registries, TAVI has transformed the structural heart disease management landscape. Despite its widespread adoption, optimal perioperative anticoagulation management remains a subject of intense debate and research [2]. Current guidelines offer limited clarity, leaving clinicians to navigate the delicate balance between thromboembolic and bleeding risks in this complex patient population [3].

Oral anticoagulation is a cornerstone of treatment for many patients undergoing TAVI, often due to pre-existing conditions such as atrial fibrillation or mechanical heart valves [4]. The decision to continue or interrupt this therapy during the procedure carries significant implications for patient outcomes, particularly regarding the risks of thromboembolic and bleeding events. On one hand, continuing anticoagulation may reduce the risk of ischemic stroke and other thromboembolic complications, such as valve thrombosis or systemic embolism. On the other hand, it may increase the likelihood of life-threatening bleeding, including pericardial tamponade, access-site hemorrhage, or gastrointestinal bleeding. Striking the right balance between these competing risks is crucial, as both types of events can significantly impact morbidity, mortality, and long-term recovery [4-5]. Recent meta-analyses have shed light on the efficacy and safety profiles of various anticoagulation strategies in the context of TAVI [6-7]. These studies have compared direct oral anticoagulants (DOACs) with traditional vitamin K antagonists (VKAs), as well as single antiplatelet therapy (SAPT) versus dual antiplatelet therapy (DAPT). The findings from these analyses provide crucial insights into the optimal management of anticoagulation in TAVI patients. 

One significant aspect of this research is the evaluation of major bleeding risks associated with different anticoagulation regimens. A comprehensive network meta-analysis of randomized controlled trials (RCTs) found that SAPT more than halved the risk of bleeding compared to DAPT- and DOAC-based regimens in patients without an indication for chronic oral anticoagulation [6]. This finding highlights the importance of tailoring anticoagulation strategies to individual patient risk profiles. 

The comparison between DOACs and VKAs has also been a focus of recent meta-analyses. These studies aim to determine the comparative efficacy and safety of these two classes of anticoagulants in TAVI patients. The results of these analyses contribute to the ongoing refinement of anticoagulation protocols in the perioperative management of TAVI [8]. Furthermore, the timing of anticoagulation initiation or re-initiation post-TAVI is another critical factor under investigation. The timing of anticoagulation initiation post-TAVI remains controversial, as early initiation reduces thromboembolic risks like valve thrombosis but increases bleeding complications. Delayed initiation minimizes bleeding risks but may leave patients vulnerable to early thromboembolic events [5-6]. Some studies have explored the use of non-vitamin K oral anticoagulants (NOACs) after TAVI, assessing their efficacy and safety compared to traditional anticoagulation strategies [9]. 

As the field of TAVI continues to evolve, so too does our understanding of optimal anticoagulation management. The complex interplay between patient factors, procedural considerations, and pharmacological interventions necessitates a nuanced approach to anticoagulation during TAVI. This introduction sets the stage for a comprehensive meta-analysis examining the continuation versus interruption of oral anticoagulation during TAVI, a topic of significant clinical relevance and ongoing research interest. By synthesizing data from multiple studies and leveraging advanced statistical techniques, this meta-analysis aims to provide a robust evidence base to guide clinical decision-making. The findings will contribute to the development of standardized protocols for anticoagulation management in TAVI patients, potentially improving outcomes and reducing complications associated with this increasingly common procedure.

## Review

Methodology 

Literature Search 

A comprehensive literature search was performed using multiple electronic databases, including PubMed, Embase, Cochrane Library, and Web of Science. The search strategy employed a combination of Medical Subject Headings (MeSH) terms and keywords related to TAVI, oral anticoagulation, and perioperative management. The search was limited to English-language publications from inception to November 2024. Additionally, reference lists of relevant articles and reviews were manually screened to identify any missed studies. We also explored clinicaltrial.gov to explore recently conducted trials on this topic. 

Study Selection 

Two independent reviewers screened the titles and abstracts of the retrieved articles for eligibility. Studies were included based on predefined criteria: RCTs or observational studies, adult patients undergoing TAVI, comparison of continuation versus interruption of oral anticoagulation during TAVI, and reporting at least one of the predefined outcomes. We excluded reviews, editorials, case reports, case series, and animal studies. We excluded studies lacking control or comparison groups. Full-text articles of potentially eligible studies were obtained and assessed independently by the same two reviewers. Disagreements were resolved through discussion or consultation with a third reviewer. 

Data Extraction 

Two independent reviewers extracted data using a standardized form, collecting information on study characteristics, patient demographics, anticoagulation regimens, procedural details, and predefined outcomes. The primary outcomes of interest were all-cause mortality, myocardial infarction (MI), stroke, and major bleeding. One author extracted the data and the second author cross-checked it and entered it into the software for data analysis. 

Data Analysis 

All statistical analyses were conducted using Review Manager (RevMan) version 5.4 [10]. Risk ratios (RRs) with 95% confidence intervals (CIs) were calculated for dichotomous outcomes. A two-tailed *P*-value < 0.05 was considered statistically significant, ensuring rigorous statistical interpretation of the findings. Heterogeneity was rigorously evaluated using the *I*² statistic, with values >50% indicating substantial heterogeneity. We were not able to perform publication bias as only three studies were included in this meta-analysis. 

Results 

**Figure 1:**
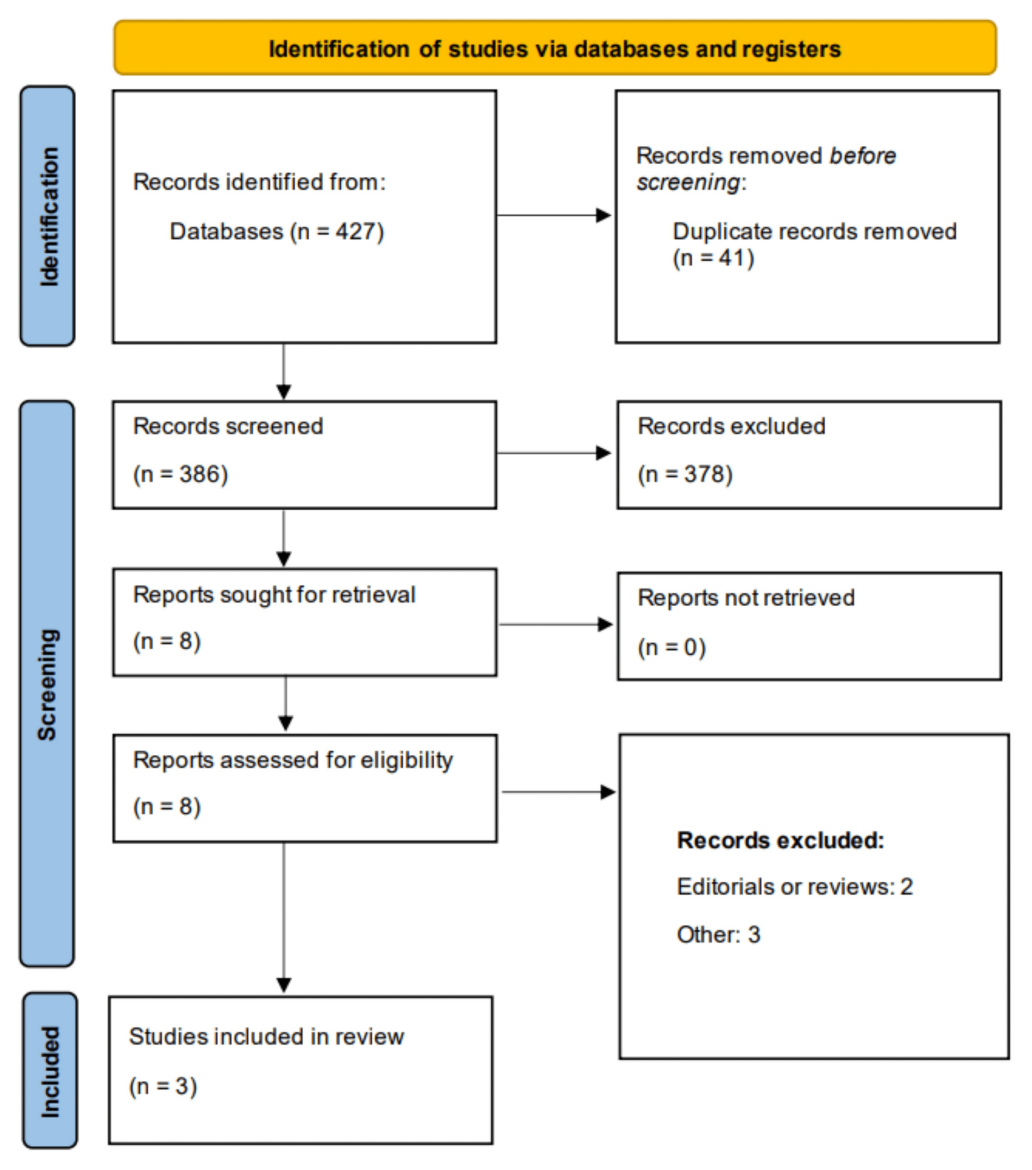
PRISMA flowchart. PRISMA, Preferred Reporting Items for Systematic Reviews and Meta-Analyses

Figure 1 shows the Preferred Reporting Items for Systematic Reviews and Meta-Analyses (PRISMA) flowchart demonstrating the study selection process. Online database screening yielded 427 studies. After removing duplicates, 386 papers were initially screened using their abstracts and titles. Through initial screening, we found eight papers relevant for full-text screening. Detailed assessment of eight papers was performed based on predefined eligibility criteria. Finally, three papers were relevant and included in this meta-analysis. Table 1 shows the characteristics of the included studies. The pooled sample was 2,591 patients, including 1,132 subjects in the continuation group and 1,459 in the interruption group. Out of three included studies two were observational, while one was RCT. All included studies have a follow-up duration of one month.

**Table 1:** Characteristics of included studies. RCT, randomized controlled trial

Author ID	Year	Study design	Groups	Sample size	Follow-up	Age (Years)	Male (*n*)
Brinkert et al. [11]	2020	Observational	Continuation Group	584	1 month	82	297
			Interruption Group	733		82	351
van Ginkel et al. [12]	2024	RCT	Continuation Group	431	1 month	81.4	273
			Interruption Group	427		80.9	289
Mangner et al. [13]	2019	Observational	Continuation Group	117	1 Month	80	46
			Interruption Group	299		80	127

All-Cause Mortality 

**Figure 2:**
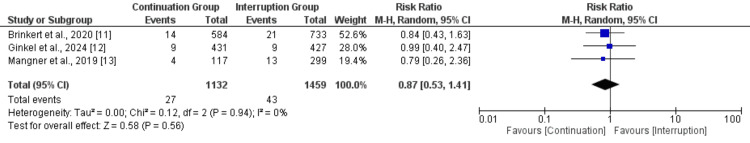
Comparison of risk of death between two groups. Sources: [11-13].

We performed a pooled analysis of three studies to compare all-cause mortality between continuous and interrupted anticoagulant groups. Figure 2 shows the findings of the pooled analysis. We did not find any significant difference in risk of all-cause mortality between continuous and interrupted anticoagulant groups (RR: 0.87, 95% CI: 0.53-1.41). No substantial heterogeneity was reported among the study results (*I*^2^: 0%).

Myocardial Infarction 

**Figure 3:**
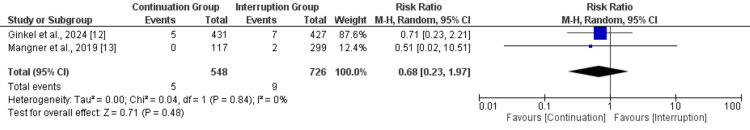
Comparison of risk of MI between two groups. Sources: [12-13]. MI, myocardial infarction

We performed a pooled analysis of two studies to compare MI between continuous and interrupted anticoagulant groups. Figure 3 shows the findings of the pooled analysis. We did not find any significant difference in the risk of MI between continuous and interrupted anticoagulant groups (RR: 0.68, 95% CI: 0.23-1.97). No substantial heterogeneity was reported among the study results (I-square: 0%). 

Stroke 

**Figure 4:**
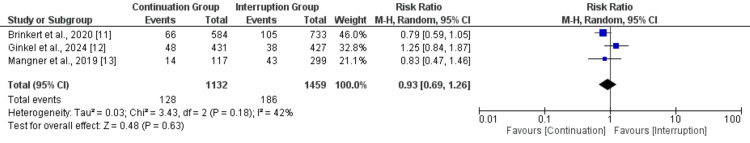
Comparison of risk of stroke between two groups Sources: References [11-13]

We performed a pooled analysis of three studies to compare the risk of stroke between continuous and interrupted anticoagulant groups. Figure 4 shows the findings of the pooled analysis. We did not find any significant difference in the risk of stroke between continuous and interrupted anticoagulant groups (RR: 0.67, 95% CI: 0.42-1.08). No substantial heterogeneity was reported among the study results (*I*^2^: 0%).

Major Bleeding 

**Figure 5:**
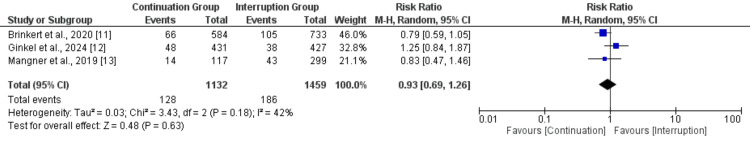
Comparison of risk of major bleeding events. Sources: [11-13].

We performed a pooled analysis of three studies to compare the risk of major bleeding between continuous and interrupted anticoagulant groups. Figure 5 shows the findings of the pooled analysis. We did not find any significant difference in the risk of major bleeding between continuous and interrupted anticoagulant groups (RR: 0.93, 95% CI: 0.69-1.26). No substantial heterogeneity was reported among the study results (*I*^2^: 42%).

Discussion 

This meta-analysis assessed the outcomes of continued versus interrupted anticoagulation strategies in patients undergoing TAVI. The results indicated that continued anticoagulation was not demonstrated to be non-inferior to interrupted anticoagulation in terms of all-cause mortality, MI, stroke, or major bleeding. To the best of our knowledge, this is the first meta-analysis to evaluate these two strategies in this patient population. We observed low heterogeneity across all outcomes, likely due to the limited number of studies and the inclusion of studies exclusively involving TAVI patients, resulting in a relatively homogeneous population.

Major and life-threatening bleeding complications remain common following TAVI, contributing to extended hospital stays, increased healthcare costs, higher morbidity, and elevated mortality rates [14-15]. The reported incidence of these events in various registries and trials has ranged from 6% to 30% [16-17]. While our analysis revealed no statistically significant difference in major bleeding events between the two anticoagulation strategies, it highlighted trends worth exploring. Two out of the three included studies, both observational, showed a slightly higher number of bleeding events in the interrupted anticoagulation group [11,13]. Conversely, a recently conducted RCT reported a higher incidence of major bleeding in the continued anticoagulation group but did not perform subgroup analysis for these events [12]. Such discrepancies underscore the potential influence of selection bias and confounding factors in observational studies. Future research, particularly large-scale randomized trials, is essential to address these limitations and clarify how these anticoagulation approaches can be tailored to specific patient populations.

Interrupting oral anticoagulation may be especially suitable for TAVI patients, given their heightened risk for procedural bleeding due to advanced age, multiple comorbidities, increased frailty, and the use of larger vascular access devices [18]. This approach could be particularly beneficial in patients with elevated bleeding risk scores or undergoing complex procedures. On the other hand, patients with higher CHA2DS2-VASc scores, indicative of elevated thromboembolic risk, may benefit from continued anticoagulation to mitigate the risks of stroke or valve thrombosis [12].

The absence of randomized trial data on the optimal periprocedural oral anticoagulation strategy for patients undergoing TAVI results in significant variability in clinical practices [18]. While some centers opt to interrupt oral anticoagulation for varying lengths of time, others maintain anticoagulation throughout the periprocedural period. This choice often depends on the type of oral anticoagulant being used. DOACs are typically interrupted due to their rapid onset and offset, which allows for predictable management, while VKAs are more commonly continued, given their longer half-life and the availability of established reversal agents [19]. These pharmacokinetic differences may also influence safety profiles, as DOACs could potentially be safer for specific bleeding complications, such as access-site hematomas, in this population. However, the limited availability of DOAC reversal agents at many centers may lead to a preference for VKAs in certain clinical scenarios [11,20].

This meta-analysis found a lower risk of stroke in patients who continued oral anticoagulation therapy; however, the difference was not statistically significant. All the included studies yielded comparable findings, resulting in no heterogeneity in the pooled analysis. Notably, none of the studies conducted subgroup analyses to evaluate the effectiveness of the two strategies in specific patient populations. One included trial [12], however, demonstrated that the composite outcome of all-cause mortality, MI, and stroke was higher in the continuation group among patients with a CHA2DS2-VASc score below 5. This finding suggests that continued oral anticoagulation may be particularly beneficial for patients with higher CHA2DS2-VASc scores or a history of stroke, as these individuals face an elevated risk of thromboembolic complications due to underlying vascular or cerebrovascular disease. Such findings highlight the importance of individualized anticoagulation strategies based on thrombotic and bleeding risk assessments.

The lack of long-term follow-up in the included studies may obscure critical differences in thromboembolic or bleeding events, which often occur beyond the one-month timeframe. CIs and the magnitude of risk differences, even if not statistically significant, remain clinically relevant and warrant consideration in decision-making. Future research should focus on larger RCTs with subgroup analyses based on bleeding and thromboembolic risk scores, procedural complexity, and anticoagulant types to guide personalized management. In the meantime, clinicians might consider interruption of anticoagulation for patients at high bleeding risk or undergoing complex procedures, while continuation may be prioritized for those at high thromboembolic risk, particularly those with CHA2DS2-VASc scores above 5.

The present meta-analysis has several important limitations. Firstly, only three studies were included, with just one being an RCT, limiting the robustness of our findings. Secondly, the lack of sufficient data prevented subgroup analyses, which could have provided insights into the efficacy of anticoagulation strategies across different patient populations. Thirdly, the short-term nature of the follow-up data, limited to one month in all studies, restricts our understanding of the long-term impacts of continued versus interrupted anticoagulation strategies. This is particularly significant given that TAVI patients often require long-term management of both their valvular condition and anticoagulation needs. Lastly, we were not able to perform publication bias due to a limited number of studies. These limitations highlight the need for larger, well-designed studies with extended follow-up periods. 

This meta-analysis highlights the need for further research to optimize anticoagulation strategies in TAVI patients. Future studies should focus on identifying patient subgroups that may benefit from continued or interrupted anticoagulation, particularly considering factors such as CHA2DS2-VASc scores and bleeding risk. Larger RCTs are necessary to provide more definitive evidence on the safety and efficacy of different anticoagulation approaches. Clinically, the findings suggest that individualized anticoagulation strategies may be necessary. For patients at high risk of thromboembolic events, continued anticoagulation might be preferred, while those with elevated bleeding risk may benefit from interruption. The choice between VKAs and DOACs should also be considered, given their different pharmacokinetic profiles and the availability of reversal agents [10,18]. These results underscore the importance of careful risk stratification and personalized decision-making in TAVI patients requiring anticoagulation.

## Conclusions

This meta-analysis of three studies, including 2,591 patients, found no significant differences between continuation and interruption of oral anticoagulation during TAVI in terms of all-cause mortality, MI, stroke, or major bleeding. While continued anticoagulation showed a trend toward lower stroke risk, this difference was not statistically significant. Given the limited number of studies and short follow-up periods, larger RCTs are needed to definitively establish the optimal anticoagulation strategy. Currently, the choice between continuation and interruption of anticoagulation should be individualized based on patient-specific factors, particularly considering thromboembolic and bleeding risks.
